# An impaired intrinsic microglial clock system induces neuroinflammatory alterations in the early stage of amyloid precursor protein knock-in mouse brain

**DOI:** 10.1186/s12974-019-1562-9

**Published:** 2019-08-30

**Authors:** Junjun Ni, Zhou Wu, Jie Meng, Takashi Saito, Takaomi C. Saido, Hong Qing, Hiroshi Nakanishi

**Affiliations:** 1 Department of Aging Science and Pharmacology, Faculty of Dental Sciences, Kyushu University, Fukuoka, 812-8582 Japan; 2 Laboratory for Proteolytic Neuroscience, RIKEN Brain Science Institute, Wako, 351-0198 Japan; 3 Key Laboratory of Molecular Medicine and Biotherapy, School of Life Science, Beijing Institute of Technology, Haidian District, Beijing, 100081 People’s Republic of China; 4 Department of Pharmacology, Faculty of Pharmacy, Yasuda Women’s University, Hiroshima, 731-0153 Japan

**Keywords:** Clock genes, BMAL1, Microglia, APP-KI mice, Neuroinflammation, Alzheimer’s disease

## Abstract

**Background:**

Disturbances in clock genes affect almost all patients with Alzheimer’s disease (AD), as evidenced by their altered sleep/wake cycle, thermoregulation, and exacerbation of cognitive impairment. As microglia-mediated neuroinflammation proved to be a driver of AD rather than a result of the disease, in this study, we evaluated the relationship between clock gene disturbance and neuroinflammation in microglia and their contribution to the onset of AD.

**Methods:**

In this study, the expression of clock genes and inflammatory-related genes was examined in MACS microglia isolated from 2-month-old amyloid precursor protein knock-in (APP-KI) and wild-type (WT) mice using cap analysis gene expression (CAGE) deep sequencing and RT-PCR. The effects of clock gene disturbance on neuroinflammation and relevant memory changes were examined in 2-month-old APP-KI and WT mice after injection with SR9009 (a synthetic agonist for REV-ERB). The microglia morphology was studied by staining, neuroinflammation was examined by Western blotting, and cognitive changes were examined by Y-maze and novel object recognition tests.

**Results:**

CLOCK/BMAL1-driven transcriptional negative feedback loops were impaired in the microglia from 2-month-old APP-KI mice. Pro-inflammatory genes in microglia isolated from APP-KI mice were significantly higher than those isolated from WT mice at Zeitgeber time 14. The expression of pro-inflammatory genes was positively associated with NF-κB activation and negatively associated with the BMAL1 expression. SR9009 induced the activation of microglia, the increased expression of pro-inflammatory genes, and cognitive decline in 2-month-old APP-KI mice.

**Conclusion:**

Clock gene disturbance in microglia is involved in the early onset of AD through the induction of chronic neuroinflammation, which may be a new target for preventing or slowing AD.

**Electronic supplementary material:**

The online version of this article (10.1186/s12974-019-1562-9) contains supplementary material, which is available to authorized users.

## Background

McGeer and McGeer [1] suggested the pathological importance of neuroinflammation mediated by microglia rather than amyloid β (Aβ) in the pathogenesis of Alzheimer’s disease (AD), as anti-inflammatory agents have a substantial sparing effect on AD [2]. Although the neuroinflammation regularly observed in AD has long been incorporated into the amyloid cascade hypothesis of AD, two recent reports have suggested an inflammatory hypothesis for AD. Treatment with the colony-stimulating factor 1 receptor (CSF1R) inhibitors, that block microglial proliferation and rescue inflammatory alterations, improved the performance in memory and behavioral tasks without affecting the accumulation of Aβ in mouse models of AD [3, 4]. Furthermore, previous studies have provided evidence that infection of the brain with microbes is linked to AD [5, 6]. Some microbes can remain latent in the nervous system with the potential for reactivation, and neuronal damage caused by direct microbial action and microbe-induced inflammation might occur years after the initial infection. In line with this infection hypothesis of AD, we previously reported that virulence factors of *Porphylomonas gingivalis*, a major pathogen in periodontal disease, including lipopolysaccharide (LPS) and gingipains, were able to activate microglia to induce neuroinflammation through the activation of Toll-like receptor 2 and protease-activated receptor-2, respectively, [7, 8]. These observations suggest that excessive neuroinflammation mediated by microglia is a key driver of AD rather than a result of the disease.

Circadian rhythm disturbances have long been considered a consequence of neurodegeneration associated with AD. AD patients exhibit profound disruptions in their circadian rhythm concerning sleep-wakefulness and other processes. In the post-mortem brain tissue of AD patients, altered synchronization in the rhythms of clock gene expression, including period 1 (*PER1*), period 2 (*PER2*), and brain and muscle Arnt-like protein-1 (*BMAL1*), was noted in different regions of AD patients compared with control subjects [9]. The clock gene system is known to be capable of influencing the inflammatory process in a number of ways. BMAL1 is the central mediator of the circadian control of the immune system and promotes an anti-inflammatory state [10]. A recent clinical study showed that the significant deregulation of *BMAL1* in the brain was associated with early AD [11]. An intrinsic molecular clock also exists in microglia that control diurnal morphological changes in their processes, and these cells regulate the sleep-wake cycle-dependent changes in synaptic strengths [12–14]. Furthermore, infection of *P. gingivalis* induced process extension of cortical microglia, and this response was significantly greater during the day than night [15], suggesting that the intrinsic microglial clock limits the over-reaction and inflammatory responses of microglia during the active phase. However, little is known about the possible contribution of microglial intrinsic molecular clock to the neuroinflammatory response in AD pathology.

Although a number of AD mouse models have been developed based on amyloid precursor protein (APP) overexpression, the overexpression paradigm may cause additional phenotypes unrelated to AD, including the overproduction of soluble N-terminal fragments, C-terminal fragment-α, C-terminal fragment-β, and APP intracellular fragments [16]. To overcome these drawbacks, two novel AD mouse models (single humanized APP knock-in (KI) mice carrying Swedish (NL), Beyreuther/Iberian (F), or Arctic (G) mutations in different combinations) were generated by knock-in (KI) of a humanized Aβ sequence bearing AD-associated mutations into the mouse APP locus [17]. These models exhibit unique pathophysiologic properties in the brain. For example, APP-KI^NL-G-F/NL-G-F^ mice, which bear all three mutations, show aggressive Aβ pathology starting at 2 months of age [18].

We showed for the first time in the present study that a reduced expression level of BMAL1 was responsible for the increase in the inflammatory phenotype of microglia in APP-KI mice through a reduction in RORα, which in turn reduced IκBα and enhanced NF-κB activation. To determine the pathological roles of REV-ERB in the inflammatory response and learning ability of APP-KI mice, we evaluated the effects of SR9009, a synthetic agonist for REV-ERB, because REV-ERBα nuclear receptor plays a pivotal role in the negative feedback loop regulating the *BMAL1* and *CLOCK* expression. These results suggest that an impaired intrinsic microglial clock system contributes to neuroinflammatory responses and the resultant cognitive impairment in the early stage of AD.

## Methods

### Animals

Wild-type (WT) and APP-KI mice on a C57BL/6 background were kept in a specific pathogen-free environment at Kyushu University Faculty of Dental Science. Under light-dark conditions, the Zeitgeber time 0 (ZT0) was designated as lights on and ZT12 as lights off. The line of APP-KI mice carried the Arctic mutation, Swedish, and Beyreuther/Iberian mutations. The selection of APP-KI homozygous mice from their littermates obtained by heterozygous coupling was performed by examining the template genomic DNA isolated from tail biopsies, using primers 5′-ATCTCGGAAGTGAAGATG-3′, 5′-ATCTCGGAAGTGAATCTA-3′, 5′-TGTAGA TGAGAACTTAAC-3′, and 5′-CGTATAATGTATGCTATACGAAG-3′. Male mice were used in the whole study. Two-month-old WT and APP-KI mice were administered SR9009 (Millipore) 100 mg/kg (intraperitoneally) for 14 days. All animal experiments were conducted in accordance with the guidelines contained in the Act on Welfare and Management of Animals (Ministry of Environment of Japan) and Regulation of Laboratory Animals (Kyushu University) and under the protocols approved by the Institutional Animal Care and Use Committee review panels at Kyushu University.

### Immunoblotting analyses

The brain cortical tissues were collected from APP-KI mice with or without SR9009 injection. The brain lysates were prepared as described previously [23]. The following primary antibodies were used: rabbit anti-Iba1 (1:1000; WAKO), mouse anti-NOS2 (1:1000; Abcam), mouse anti-IL-1β (1:1000, Santa Cruz Biotechnology), mouse anti-actin (1:5000; Abcam), mouse anti-phospho-IκBα (1:1000, Santa Cruz Biotechnology), rabbit anti-IκBα (1:1000, Santa Cruz Biotechnology), and mouse anti-Aβ (6E10, 1:1000, Covance). The following were used as secondary antibodies: horseradish peroxidase (HRP)-labeled anti-rabbit (1:2000; GE Healthcare) and anti-mouse (1:2000; R&D Systems). The HRP-labeled antibodies were detected using an enhanced chemiluminescence detection system (ECL Kit; GE Healthcare) with an image analyzer (LAS-1000; Fuji Photo Film).

### Real-time polymerase chain reaction (PCR)

The mRNA isolated from the isolated microglia of each group at different time points were subjected to a real-time quantitative RT-PCR. The total RNA was extracted with the RNAiso Plus (Takarada, Japan) according to the manufacturer’s instructions. A total of 1000 ng of extracted RNA was reverse transcribed to cDNA using the QuantiTect Reverse Transcription Kit (Qiagen, Japan). After an initial denaturation step at 95 °C for 5 min, temperature cycling was initiated. Each cycle consisted of denaturation at 95 °C for 5 s, annealing at 60 °C for 10 s, and elongation for 30 s. In total, 40 cycles were performed. The cDNA was amplified in duplicate using a Rotor-Gene SYBR Green RT-PCR Kit (Qiagen, Japan) with a Corbett Rotor-Gene RG-3000A Real-Time PCR System. The data were evaluated using the RG-3000A software program (version Rotor-Gene 6.1.93, Corbett). The sequences of primer pairs were as follows:

BMAL1: 5′-CTATCTTCCTCGGACACTGC-3′ and 5′-CTTCTTGCCTCCTGGAGAAG-3′; PER1: 5′-CCAGATTGGTGGAGGTTACTGAGT-3′ and 5′-GCGAGAGTCTTCTTGGAGCAGTAG-3′; PER2: 5′-TTCCACTATGTGACAGCGGAGG-3′ and 5′-CGTATCCAT TCATGTCGGGCTC-3′; REV-ERBα: 5′-CCCTGGACTCCAATAACAACACA-3′ and 5′-GCCATTGGAGCTGTCACTGTAG-3′; TNF-α: 5′-CTGTAGCCCACGTCGTAGC-3′ and 5′-TTGAGATCCATGCCGTTG-3′; IL-1β: 5′-CAACCAACAAGTGATATTCTCCATG-3′ and 5′-GATCCACACTCTCCAGCTGCA -3′; NOS2: 5′-GCCACCAACAATGGCAAC-3′ and 5′-CGTACCGGATGAGCTGTGAATT-3′; IL-6: 5′-TCAATTCCAGAAACCGCT ATGA-3′ and 5′-CACCAGCATCAGTCCCAAGA-3′; RORα: 5′-TTCTAAAAGCAGGCT CGCTAGAG-3′ and 5′-AAGTACACGGTGTTGTTCTGAGAGTC-3′; IκBα: 5′- GAAGCCGCTGACCATGGAA-3′ and 5′-GATCACAGCCAAGTGGAGTGGA-3′; Actin: 5′-AGAGGGAAATCGTGCGTGAC-3′ and 5′-CAATAGTGATGACCTGGCCGT-3′.

For data normalization, an endogenous control (actin) was assessed to control for the cDNA input, and the relative units were calculated by a comparative Ct method. All of the real-time RT-PCR experiments were repeated three times, and the results are presented as the means of the ratios ± standard error of the mean (SEM).

### Cell isolation

CD11b^+^ microglial cells were isolated from the mouse brain by the magnetic cell sorting (MACS) method as described previously [19]. Mice in each group were anesthetized and transcardially perfused with phosphate-buffered saline (PBS). The brains were separated as contralateral and ipsilateral samples and cut into small pieces. After enzymatic digestion using a Neural Tissue Dissociation Kit (Papain), the cell suspensions were further mechanically dissociated using a gentle MACS Dissociator (Milteny Biotec, Bergisch Gladbach, Germany). The single-cell suspensions were obtained after application to a 30-mm cell strainer. After magnetic labeling with CD11b Microbeads, the cell suspension was loaded onto a magnetic column placed in the magnetic separator (Milteny Biotec). The MACS column was then rinsed with PBS, and the CD11b-positive fraction was collected.

### Cell culture

The mouse microglial cell line MG6 (Riken Cell Bank, RCB2403) was maintained in DMEM containing 10% fetal bovine serum (Gibco) supplemented with 450 mg/ml glucose (Gibco), penicillin-streptomycin (Gibco), 10 μg/ml insulin, and 100 μM β-mercaptoethanol in accordance with the previously described methods [20, 21]. They were synchronized by treatment with 100 nM dexamethasone (Wako) for 2 h and then stimulated with 10 μM SR9009 and 1 μM oligomeric Aβ (OAβ) at the indicated time points. Non-synchronizing cells were treated with 50 ng/ml LPS (Sigma) or combination with 10 μM SR9009 for 8 h.

### CAGE RNA sequencing of microglia from WT and APP-KI mice

ZT2 and ZT14 were 2 h after light turning on and turning off which can typically represent the day time and night time. Therefore, CD11b^+^ microglial cells were acutely isolated from WT and APP-KI mouse brain at ZT2 and ZT14 by MACS methods, and then, 5 μg RNA of each group was prepared for sequencing. Cap analysis gene expression (CAGE) library preparation, sequencing, mapping, and gene expression were performed by DNAFORM (Yokohama, Kanagawa, Japan). In brief, the RNA quality was assessed by a Bioanalyzer (Agilent) to ensure that the RNA integrity number (RIN) was over 7.0 and that the A260/A280 and 260/230 ratios were over 1.7. First-strand cDNAs were transcribed to the 5′ end of the capped RNAs and attached to CAGE “bar code” tags, and then, the sequenced CAGE was mapped to the mouse mm9 genomes using the BWA software program (v0.5.9) after discarding ribosomal or non-A/C/G/T base-containing RNAs. For tag clustering, the CAGE-tag 5′ coordinates were input for Reclu clustering, with a maximum irreproducible discovery rate (IDR) of 0.1 and minimum count per million (CPM) value of 0.1 [22].

### Locomotor activity

Fourteen days after SR9009 treatment, locomotor activity was examined (day 15). Mice were removed from their home cages and placed in a novel home cage (clean and without bedding), which provided a floor area of 28 × 18 cm, and then, the locomotor activity of mice of each genotype and each different age group was scored for 3 min. The novel home cage was divided into six identical rectangles and a trained observer determined the incidence of line crossing.

### Novel object recognition test

Fourteen days after SR9009 treatment, novel object recognition tests were examined (day 15–day 18). Mice were individually habituated to an open-field box (58 × 42 × 35 cm) by being given 10 min of exploration time in an empty arena for 3 days (habituation session). During the acquisition phase, two objects of the same material were placed in symmetrical positions at the center of the box for 10 min. One hour after the acquisition phase training, one of the objects was replaced with a novel object, and the exploratory behavior was again analyzed for 3 min. After each session, the objects were thoroughly cleaned with 75% ethanol to prevent odor recognition. Exploration of an object was defined as rearing on the object or sniffing it at a distance of < 1 cm, touching it with the nose, or both. Successful recognition of a previously explored object was reflected by preferential exploration of the novel object. Discrimination of spatial novelty was assessed by comparing the difference between the time spent exploration of the novel and familiar objects and the total time spent exploring both objects, which made it possible to adjust for differences in total exploration time.

### Y-maze test

Y-maze test was examined prior to Novel object recognition test on the same day (day 15). Testing occurred in a Y-shaped maze with three identical black Plexiglas arms at 120° angles from each other (40 × 10 × 20 cm; Shinfactory, Fukuoka, Japan). After being introduced to the end of one arm, the mice were allowed to freely explore the three arms in a 5-min session. The number of arm entries and the number of triads were recorded in order to calculate the percentage of alternation. The three consecutive choices were defined as an alternation. The percentage of alternations was calculated as (actual alternations/maximum alternations) × 100.

### Immunofluorescent staining

Mouse brain samples for immunofluorescent staining were prepared as previously reported [20, 21]. Sections for staining were incubated with the following primary antibodies: rabbit anti Iba1 (1:2000, Wako) and mouse anti-Aβ (6E10, 1:1000, Covance) at 4 °C overnight. After being washing with PBS, the sections were incubated with donkey anti-rabbit Alexa 488 (1:500; Jackson ImmunoResearch) and donkey anti-mouse Cy3 (1:500; Jackson ImmunoResearch) at 4 °C for 2 h. After further washing, the sections were mounted in Vectashield anti-fading medium (Vector Laboratories). The fluorescence images were observed using a confocal laser scanning microscope (CLSM; C2si, Nikon).

### Quantitative morphological analyses of microglia

Confocal Z stack images were captured from the brain of APP-KI mice with or without SR9009 injection. Somata of microglia were quantified after outlining using the ImageJ software program as reported previously [23]. The morphological analyses of microglia were performed using Z-projections of confocal images. Microglial processes were traced and reconstructed as a single microglia image using the Simple Neurite Tracer program, and the total process length was semi-automatically traced using three-dimensional image data. Single microglia with topological skeletonized images were converted using the skeletonize program.

### Statistical analysis

The data are represented as the means ± SEM. The statistical analyses were performed by Student’s *t* test or a one- or two-way analysis of variance (ANOVA) with a post hoc Tukey’s test using the GraphPad Prism software package (GraphPad Software). A value of *p* < 0.05 was considered to indicate statistical significance. The significance, phase, and amplitude of 24 h rhythms in clock gene expression were evaluated statistically using the non-parametric JTK_Cycle test implemented in R [24].

## Results

### Aberrant diurnal clock gene rhythms in cortical microglia isolated from APP-KI mice

**Fig. 1:**
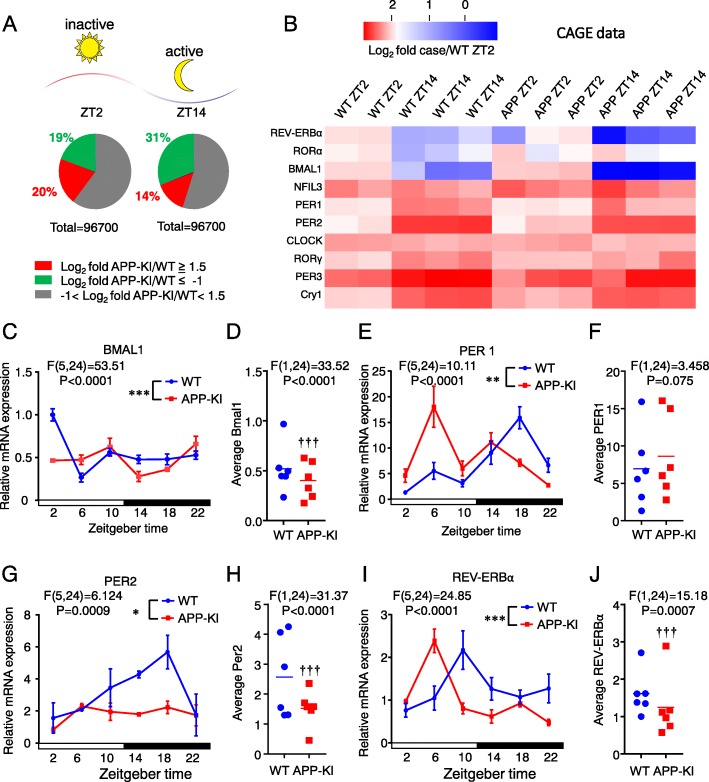
Cortical microglia isolated from 2-month-old WT and APP-KI mice showed aberrant diurnal clock gene rhythms. **a** Summary of the CAGE sequencing from microglia pooled from 2-month-old WT and APP-KI mice: APP-KI resulted in the significant upregulation of 20% TSS (FDR < 0.05, Log_2_Fc ≥ 1.5) and downregulation of 19% (FDR < 0.05, Log_2_Fc ≤ − 1) at ZT2 as well as the upregulation of 14% TSS and downregulation of 31% at ZT14. **b** The circadian clock gene expression in microglia pooled from 2-month-old WT and APP-KI mice at ZT2 and ZT14. A total of ten clock genes were clustered into five color-coded modules with blue-red representation in each group. Values are represented as the Log_2_ fold case/WT ZT2 from three different experiments carried out with microglia pooled from 12 2-month-old WT and 12 2-month-old APP-KI mice. **c**, **e**, **g**, and **i** Temporal changes in the mRNA levels of clock genes in cortical microglia of 2-month-old WT and APP-KI mice. The mRNA levels of BMAL1 (**c**), PER1 (**e**), PER2 (**g**), and REV-ERBα (**i**) were quantified using real-time PCR. Samples of each time point were pooled from three to four mice. Data are represented as the mean ± SEM of three independent experiments. The asterisks indicate a statistically significant difference from the WT group (*P < 0.05, **P < 0.01, and ***P < 0.001, two-way ANOVA test, interaction between time and genotypes). **d**, **f**, **h**, and **j** The average values of BMAL1 (**d**), PER1 (**f**), PER2 (**h**), and REV-ERBα (**j**) over the course of the day were calculated. Results were further analyzed using JTK_Cycle. The daggers indicate a statistically significant difference from the WT group (^† † †^*P* < 0.001, two-way ANOVA test, interaction between genotypes)

In order to clarify the mechanisms underlying the effects of diurnal clock genes on the pathology of AD, we performed CAGE RNA sequencing of microglia from a 2-month-old WT and APP-KI mice at ZT2 and ZT14. We identified 96,700 transcriptional start sites (TSS), 20% of which were upregulated (false discovery rage, FDR < 0.05, Log_2_Fc ≥ 1.5) and 19% of which were downregulated (FDR < 0.05, Log_2_Fc ≥ 1.5) at ZT2, 14% of which were upregulated, and 31% of which were downregulated at ZT14 in microglia from APP-KI mice compared to WT mice (Fig. 1a). A gene expression heat map with blue-red representation of 10 clock genes obtained by the CAGE method was obtained for cortical microglia isolated from WT and APP-KI mice at ZT2 and ZT14 (Fig. 1b). Both *REV-ERB* and *BMAL1* were under-expressed in cortical microglia isolated from 2-month-old WT and APP-KI mice at ZT14 relative to ZT2. In contrast, *PER1* and *PER2* were over-expressed in cortical microglia of both genetic groups at ZT14 relative to ZT2.

To verify these findings obtained by the CAGE method, the diurnal changes in the mean mRNA expression levels of *BMAL1*, *REV-ERBα*, *PER1*, and *PER2* in cortical microglia prepared from WT and APP-KI mice were examined using quantitative PCR (qPCR). As reported previously [12], *BMAL1* and *REV-ERBα* exhibited oscillations in cortical microglia isolated from WT mice with peaks at ZT2 and ZT10, respectively, (Fig. 1c, i). It is noted that the average values of mRNA expression of *BMAL1* and *REV-ERBα* over the course of the day in APP-KI microglia were significantly lower than those in WT microglia (Fig. 1d, j). The WT cortical microglia also exhibited an oscillation of *PER1* and *PER2* with a peak at ZT18. In contrast, the expression of these clock genes in cortical microglia isolated from APP-KI mice showed differential oscillations as compared to those in WT microglia (Fig. 1e, g). *BMAL1* and *REV-ERBα* exhibited oscillations with peaks at ZT22 and ZT6, respectively, whereas *PER1* exhibited an oscillation with a peak at ZT6, and *PER2* did not show a clear oscillation at all. The average value of mRNA expression of *PER2* over the course of the day in APP-KI microglia was significantly lower than that in WT microglia, while there was no significant difference in the average value of mRNA expression of *PER1* between the two groups (Fig. 1f, h). The rhythmicity in clock genes expression in microglia was further analyzed by JTK_Cycle analysis (Additional file 1: Table S1). We observed adjusted *p* value (ADJ.P) in PER1 of both WT and APP-KI and that in PER2 of WT mice showed < 0.05. However, the ADJ. P in APP-KI mice were all increased compared to that of WT mice in all the analyzed clock genes (Additional file 1: Table S1). Taken together, these results suggest that the CLOCK/BMAL1-driven transcriptional negative feedback loops are impaired in the cortical microglia of APP-KI mice.

### The comparative expression of inflammatory-related genes by microglia prepared from WT and APP-KI mice

**Fig. 2:**
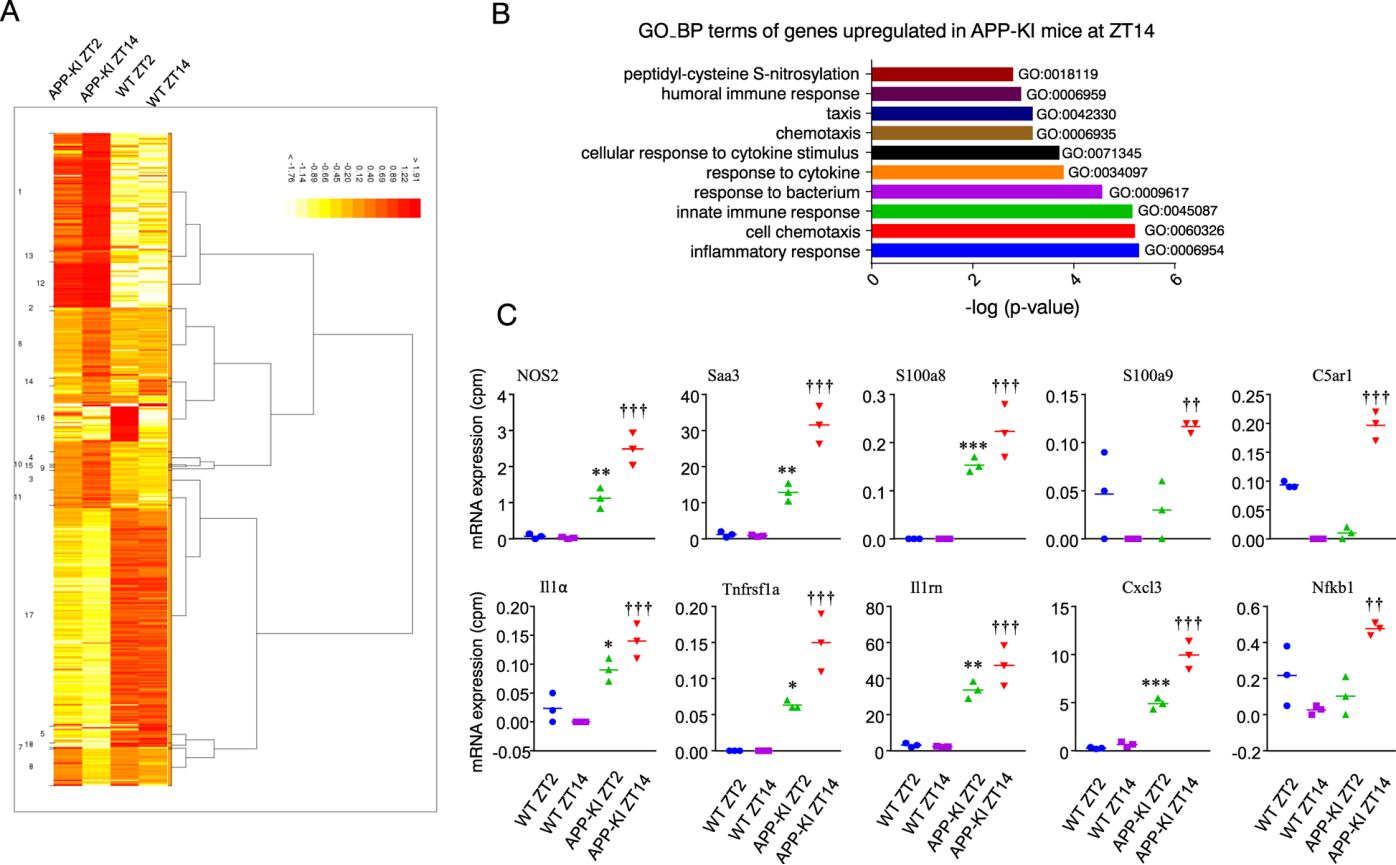
The comparative expression of the microglial inflammatory-related genes. **a** Heat map and hierarchal clustering of the transcripts in cortical microglia at ZT2 and ZT14 of both WT and APP-KI mice. **b** A gene ontology analysis of genes associated with upregulated TTS in microglia from APP-KI mice at ZT14. The ten most significantly enriched biologic process (GO-BP) terms are shown. **c** The top ten transcripts in the cluster of “inflammatory response” in cortical microglia at ZT2 and ZT14 of both WT and APP-KI mice. Values are represented as counts per million (cpm) of three independent experiments carried out with microglia isolated from 12 2-month-old WT and 12 APP-KI mice.). The asterisks indicate a statistically significant difference from the WT ZT2 group (*P < 0.05, **P < 0.01, and ***P < 0.001, Student’s *t* test). The daggers indicate a statistically significant difference from the APP-KI ZT2 group (^† †^*P* < 0.01 and ^† † †^*P* < 0.001, Student’s *t* test)

To analyze the mRNA expression profiles of cortical microglia, we additionally used CAGE method for a gene ontology analysis. Figure 2a shows the heat map and hierarchal clustering of the transcripts in cortical microglia isolated from both WT and APP-KI mice at ZT2 and ZT14. The mRNA expression profile differed markedly between the cortical microglia isolated from different genetic groups. Interestingly, the gene ontology analysis indicated that the upregulated transcripts were significantly enriched for the inflammatory response, cell chemotaxis, and immune response in microglia from APP-KI relative to WT mice at ZT14 (Fig. 2b). The top 10 transcripts belonged to families involved in immune response as follows: *nitric oxide synthase 2* (*NOS2*), *serum amyloid A3* (*Saa3*), *S100 calcium-binding protein A8* (*S100a8*), *S100 calcium-binding protein A9* (*S100a9*), *complement component 5a receptor 1* (*C5ar1*), *Interleukin 1α* (*II1α)*, *Interleukin 1 receptor antagonist* (*II1rn)*, *tumor necrosis factor receptor superfamily member 1A* (*Tnfrsf1a*), *C-X-C motif chemokine ligand 3* (*Cxcl3*), and *nuclear factor-κB1* (*Nfκb1*). In addition, the mean mRNA expression levels of these genes were significantly higher in cortical microglia isolated from APP-KI mice than in those from WT mice. Furthermore, the mean mRNA expression of these genes in cortical microglia isolated from APP-KI mice at ZT14 was significantly higher than those isolated at ZT2 (Fig. 2c).

### Diurnal changes in the expression of inflammatory factors in cortical microglia isolated from WT and APP-KI mice

**Fig. 3:**
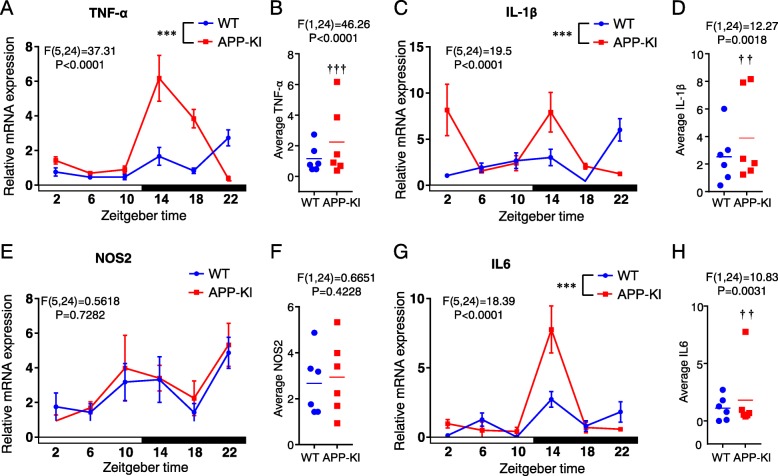
Diurnal changes of inflammatory factors in cortical microglia from 2-month-old WT and APP-KI mice. **a**, **c**, **e**, and **g** The mRNA levels of TNF-α (**a**), IL-1β (**c**), NOS2 (**e**), and IL-6 (**g**) were quantified using real-time PCR. Samples of each time point were pooled from three to four mice. Data are represented as the mean ± SEM of three independent experiments. The asterisks indicate a statistically significant difference from the WT group (***P < 0.001, two-way ANOVA test, interaction between time and genotypes). **b**, **d**, **f**, and **h** Average values of TNF-α (**a**), IL-1β (**c**), NOS2 (**e**), and IL6 (**g**) collapsed over the course of the day were calculated. Samples of each time point were pooled from three to four mice. Data are represented as mean ± SEM of three independent experiments. The daggers indicate a statistically significant difference from the WT group (^† †^*P* < 0.01, ^† † †^*P* < 0.001, two-way ANOVA test, interaction between genotypes)

To validate the data obtained by the CAGE method, we quantified the mRNA expression of *TNF-α*, *IL-1β*, *NOS2*, and *IL-6* in cortical microglia isolated from WT and APP-KI mice using qPCR. The expression of *TNF-α*, *IL-1β*, and *IL-6* in cortical microglia isolated from APP-KI mice showed differential oscillations compared with those from WT microglia (Fig. 3a, c, g). In addition, *TNF-α*, *IL-1β*, and *IL-6* in cortical microglia isolated from WT mice exhibited oscillations with peaks at ZT22, whereas their time of expression peak in microglia isolated from APP-KI mice was ZT14. No marked difference in the diurnal oscillation of the *NOS2* expression was noted between the two groups (Fig. 3e). Furthermore, there was a significant increase in the average values of mRNA expression of *TNF-α*, *IL-1β*, and *IL-6* over the course of the day in cortical microglia prepared from APP-KI mice as compared with those prepared from WT mice, but no difference was observed in the average value of mRNA expression of *NOS2* between the two groups (Fig. 3b, d, f, h).

### Diurnal changes in the mRNA expression of IκBα and RORα in cortical microglia isolated from WT and APP-KI mice

**Fig. 4:**
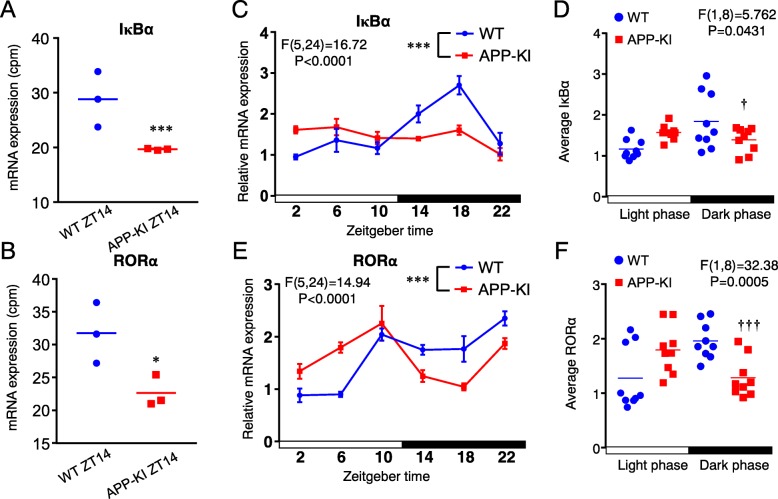
Diurnal changes of IκBα and RORα in cortical microglia isolated from WT and APP-KI mice. **a**, **b** The mRNA level of IκBα (**a**) and RORα (**b**) in cortical microglia of both WT and APP-KI 2-month old mice at ZT14 were examined by CAGE sequencing. Samples of each time point were pooled from 12 mice. The median (bold line) represent the mean ± SEM of three independent experiments (*P < 0.05, ***P < 0.001, Student’s *t* test). **c** Temporal changes in the mRNA levels of IκBα in cortical microglia of 2-month-old WT and APP-KI mice were quantified using real-time PCR. Samples of each time point were pooled from three to four mice. Data are represented as mean ± SEM of three independent experiments. The asterisks indicate a statistically significant difference from the WT group (***P < 0.001, two-way ANOVA test, interaction between time and phenotypes). **d** The average values of IκBα collapsed over the course of light phase and dark phase were calculated. Data are represented as the mean ± SEM of three independent experiments. The daggers indicate a statistically significant difference from the WT group in dark phase (†*P* < 0.05, two-way ANOVA test, interaction between time and phenotypes). **e** Temporal changes in the mRNA level of RORα in cortical microglia of both WT and APP-KI 2-month-old mice were quantified using real-time PCR. Samples of each time point were pooled from three to four mice. Data are represented as mean ± SEM of three independent experiments. The asterisks indicate a statistically significant difference from the WT group (***P < 0.001, two-way ANOVA test, interaction between time and phenotypes). **f** The average values of RORα collapsed over the course of light phase and dark phase were calculated. Data are represented as the mean ± SEM of three independent experiments. The daggers indicate a statistically significant difference from the WT group in dark phase (†††*P* < 0.001, two-way ANOVA test, interaction between time and phenotypes)

BMAL1 is known to drive the expression of RORα and thereby increase the transcription of IκBα, a major negative regulator of NF-κB. Therefore, we examined the possible diurnal changes in the mRNA expression of *IκBα* and *RORα* in cortical microglia isolated from WT and APP-KI mice. In CAGE analyses, the mean mRNA expression of *IκBα* and *RORα* in cortical microglia isolated from APP-KI mice at ZT14 was significantly lower than in those isolated from WT mice at ZT14 (Fig. 4a, b). To verify the data obtained from the CAGE analysis, we conducted qPCR analysis in cortical microglia isolated from WT and APP-KI mice. In these qPCR analyses in cortical microglia isolated from WT mice showed oscillation of *IκBα* and *RORα* with a peak at ZT18 (Fig. 4c, e). In contrast, the *IκBα* expression in cortical microglia isolated from APP-KI mice did not show a clear oscillation (Fig. 4c). The mean expression of *RORα* at ZT18 in APP-KI mice was significantly lower than that in WT mice (Fig. 4e). Furthermore, while there were no significant differences in the average values of mRNA expression of *IκBα* and *RORα* during the light phase between the two groups (Fig. 4d, f), significant differences were noted in the dark phase (Fig. 4d, f).

Given the above, we concluded that a reduced expression level of BMAL1 was responsible for the increased observation of the inflammatory phenotype of microglia in APP-KI mice through the reduction in RORα, which in turn decreased IκBα and subsequently enhanced NF-κB activation.

### Impairment of learning and memory in APP-KI mice by disturbing the intrinsic clock

REV-ERBα nuclear receptor plays a pivotal role in the negative feedback loop regulating the *BMAL1* and *CLOCK* expression [25, 26]. Therefore, it is likely that the excessive activation of REV-ERBα and subsequent reduction in the *BMAL1* expression in microglia are responsible for the altered expression profiles of inflammatory mediators in microglia of APP-KI mice. To determine the pathological role of REV-ERBα in APP-KI mice, we investigated the effects of SR9009, a synthetic agonist for REV-ERBα, on the inflammatory response and learning ability of APP-KI mice.

**Fig. 5:**
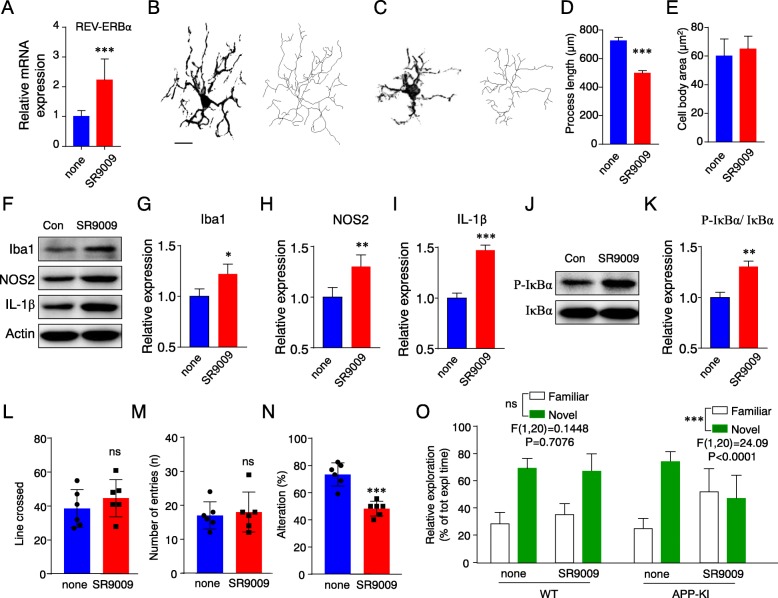
Impairment of learning and memory in APP-KI mice by disturbing the intrinsic clock. **a** The mRNA level of REV-ERBα in in the cortex from APP-KI mice with or without SR9009 injection. The results in represent the mean ± SEM of three independent experiments. The asterisks indicate a statistically significant difference from the controls (***P < 0.001, Student’s *t* test). **b**, **c** The morphological analyses of microglia in APP-KI mice without (**b**) or with SR9009 (**c**) injection. Two-dimensional (2D)-stack images of microglia were traced from CLSM images of Iba1-positive microglia. The skeletal images showing the topological staining skeletonized were reconstructed from 2D-stack images. Scale bar, 20 μm. **d** The length of the microglial process in APP-KI mice without or with SR9009 injection. **e** The size of microglial cell bodies in APP-KI mice without or with SR9009 injection. The results in **d** and **e** represent the mean ± SEM of 60–80 cells from three mice. The asterisks indicate a statistically significant difference from the controls (***P < 0.001, Student’s *t* test). **f** Immunoblots showing Iba1, NOS2, and IL-1β in the cortical lysate from APP-KI mice with or without SR9009 injection. **g-i** The quantitative analyses of Iba1, NOS2, and IL-1β in the immunoblots shown in (**f**). **j** Immunoblots showing IκBα and P-IκBα in the cortical lysate from APP-KI mice with or without SR9009 injection. **k** The quantitative analyses of Iba1, NOS2, and IL-1β in the immunoblots shown in (**j**). The results in **f-k** represent the mean ± SEM of three independent experiments. The asterisks indicate a statistically significant difference from the controls (*P < 0.05, **P < 0.01, and ***P < 0.001, Student’s *t* test). **l** The mean incidence of line-crossing. **m**, **n** The spatial working and reference memory was evaluated by the Y-maze test in APP-KI mice with or without SR9009 injection. The total number of entries of each group was calculated (**m**), and the spontaneous alternation percentages for each group were also calculated (**n**). The results are represented as the mean ± SEM (control mice, *n* = 6; SR9009 mice, *n* = 6), and the asterisks indicate a statistically significant difference from the control group (***P < 0.001, Student’s *t* test). **o** Time spent exploring the familiar and the novel objects in the recognition trial. The results are represented as the mean ± SEM (WT control mice, *n* = 6; WT SR9009 mice, *n* = 6; APP-KI control mice, *n* = 6; APP-KI SR9009 mice, *n* = 6), and the asterisks indicate a statistically significant difference from the APP-KI control group (***P < 0.001, two-way ANOVA test, interaction between two factors)

A significant increase of REV-ERBα mRNA was detected in the cortex of APP-KI after treatment with SR9009 (Fig. 5a). Next, we examined the effects of SR9009 on the activation of microglia in APP-KI mice. The morphological changes in the microglia in the hippocampus of APP-KI mice were quantitatively analyzed before and after treatment with SR9009. Confocal laser scanning microscope (CLSM) images for Iba1 were traced as stack images and then reconstructed as skeletonized images of hippocampal microglia (Fig. 5b, c). These skeletonized images of hippocampal microglia revealed a significant shortening of their processes and no change of cell body size after treatment with RS9009 (Fig. 5d, e). Furthermore, the mean protein levels of Iba1, NOS2, and mature IL-1β in the hippocampus of APP-KI mice were significantly increased after treatment with SR9009 (Fig. 5f–i). At the same time, the mean phosphorylation level of IκBα in the hippocampus of APP-KI mice was also significantly increased after treatment with SR9009 (Fig. 5j, k).

The spontaneous locomotor activity was measured in APP-KI mice with or without SR9009 injection. SR9009 injection induced no significant change in the mean number of line-crossing in APP-KI mice (Fig. 5l). We then examined the effects of SR9009 on the cognitive ability using the Y-maze and novel object recognition tests, which are simple, commonly used tests for the assessing hippocampus-dependent learning and memory. Treatment with SR9009 induced no significant change in the mean number of entries into each arm in APP-KI mice (Fig. 5m). However, APP-KI mice treated with SR9009 showed a significantly lower percentage of alternations than the untreated mice (Fig. 5n). The WT and APP-KI mice showed a response to the novel object and were able to discern a change in the object (Fig. 5o). The same results were obtained in WT mice even after treatment with SR9009 (Fig. 5o). In contrast, APP-KI mice did not show a response and could not discern a change in the object after treatment with SR9009 (Fig. 5o).

### SR9009 aggravates the APP expression and soluble Aβ production in 2-month-old APP-KI mice

**Fig. 6:**
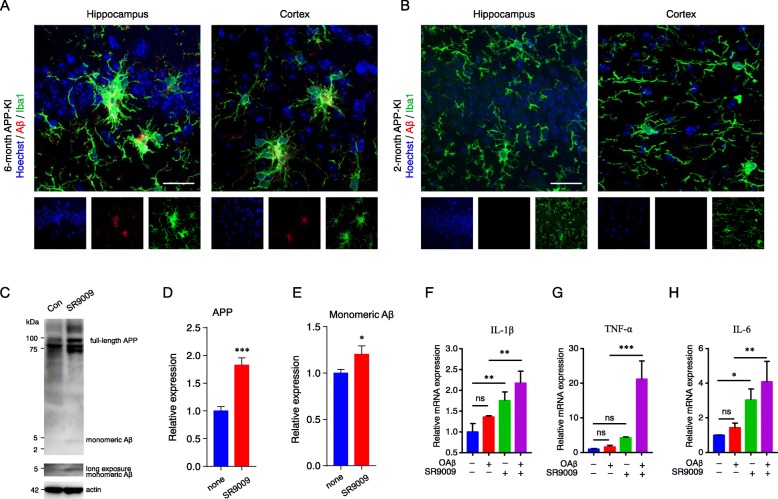
SR9009 aggravates the APP expression and soluble Aβ production in 2-month-old APP-KI mice. **a**, **b** CLSM images of the Aβ (red) merged images with Iba1 (green) in the hippocampus and cortex of 6-month-old (**a**) and 2-month-old (**b**) APP-KI mice. Scale bar, 30 μm. **c** Immunoblots showing full-length APP and monomeric Aβ in the cortical lysate from 2-month-old APP-KI mice with or without SR9009 injection. **d**, **e** The quantitative analyses of full-length APP and monomeric Aβ in the immunoblots shown in **c**. The results in **a–e** are represented as the mean ± SEM of three independent experiments. The asterisks indicate a statistically significant difference from the controls (*P < 0.05, ***P < 0.001, Student’s *t* test). **f-h** The relative mRNA levels of IL-1β (**a**), TNF-α (**b**), and IL-6 (**c**) were examined in MG6 cells 24 h after treatment with oligomeric Aβ (OAβ) alone, SR9009 alone, and their combination. The MG6 cells were synchronized by 100 nM dexamethasone (Dex) for 2 h before stimulation. Data are represented as the mean ± SEM of three independent experiments. The asterisks indicate a statistically significant difference from the indicated group (*P < 0.05, **P < 0.01, ***P < 0.001, one-way ANOVA)

To elucidate the mechanism underlying the SR9009-induced impairment of the novel object recognition in APP-KI mice, Aβ deposition and microglial activation were examined in the hippocampus and cerebral cortex of APP-KI mice. CLSM images showed that Aβ deposition and activated microglia at the site of Aβ deposition were observed in the hippocampus and cerebral cortex of 6-month-old, but not 2-month-old, APP-KI mice (Fig. 6a, b). We therefore further examined the possible deposition of monomeric Aβ in the cerebral cortex of 2-month-old APP-KI mice. Immunoblot analyses showed that treatment with SR9009 significantly increased the expression of both full-length APP and monomeric Aβ in the cortical lysate of 2-month-old APP-KI mice (Fig. 6c–e).

These effects of SR9009 in APP-KI mice prompted us to further examine a possible additive or synergistic effects of OAβ and SR9009 on microglia, as SR9009 failed to induce either an inflammatory response or cognitive impairment in WT mice. MG6 microglia were synchronized by treatment with dexamethasone and then stimulated with OAβ or combination with SR9009. Neither OAβ nor SR9009 alone influenced the mean mRNA level of TNF-α, whereas the combination of OAβ and SR9009 significantly increased the mean mRNA level of TNF-α (Fig. 6g). SR9009 alone significantly increased the mean mRNA levels of IL-1β and IL-6, whereas OAβ alone had no effect on them. However, the combination of OAβ and SR9009 further increased the mean mRNA levels of expression of IL-1β and IL-6 (Fig. 6f, h). In contrast, SR9009 significantly suppressed the mean mRNA expression levels of IL-1β and TNF-α in MG6 microglia stimulated with a high concentration of LPS (i.e., 50 ng/mL, Additional file 2: Figure S1).

### The rhythmic expression of Bma1 and REV-ERBα was impaired by OAβ in MG6 cells

**Fig. 7:**
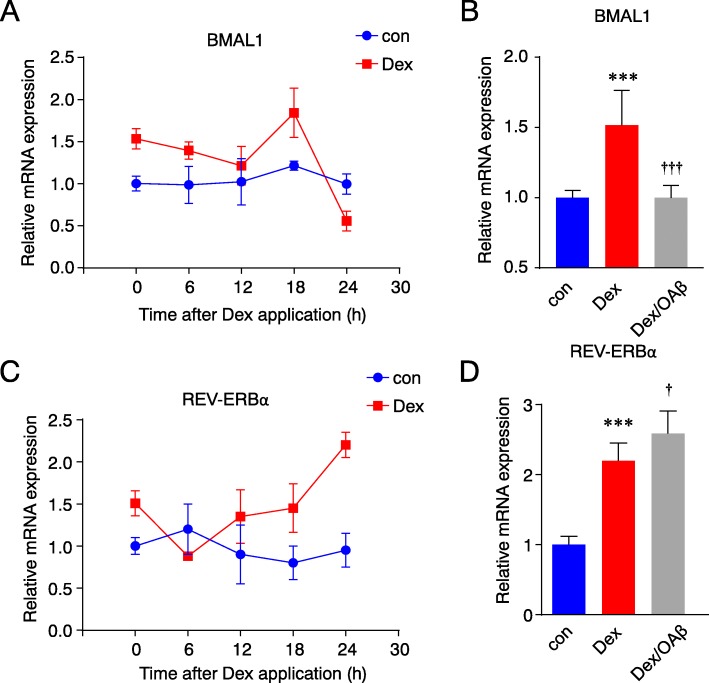
OAβ impairs the circadian clock in MG6 cells synchronized by Dex. **a**, **c** The relative mRNA levels of BMAL1 (**a**) and REV-ERBα (**c**) were examined in MG6 cells every 6 h over the course of 1 day after treatment with 100 nM Dex. **b** The relative mRNA level of BMAL1 were examined in MG6 cells 18 h after treatment with OAβ. **d** The relative mRNA levels of REV-ERBα were examined in MG6 cells 24 h after treatment with OAβ. The results in A-E are represented as the mean ± SEM of three independent experiments. The asterisks indicate a statistically significant difference from the control group (***P < 0.001, one-way ANOVA). The daggers indicate a statistically significant difference from the Dex group (^†^*P* < 0.05, ^† † †^*P* < 0.001, one-way ANOVA test)

To verify the neuroinflammation induced by combinatory treatment with OAβ and SR9009 were triggered by a disturbance in clock genes. The relative mRNA expression of Bma1 and REV-ERBα was examined through the course of a single day after synchronization by Dex. The expression of BMAL1 and REV-ERBα showed no significant changes in the control group (Fig. 7a, c). However, BMAL1 and REV-ERBα showed the rhythmic changes and peaked at 18 and 24 h, respectively, after synchronization (Fig. 7a, c). To assess the effects of OAβ on BMAL1 and REV-ERBα expression in MG6 cells, their expression was examined 18 and 24 h, respectively, after treatment with OAβ following synchronization. OAβ significantly decreased the BMAL1 expression and increased the REV-ERBα expression (Fig. 7b, d), suggesting that OAβ did indeed disturb the circadian clock, at least in MG6 cells.

## Discussion

There is increasing evidence that immune parameters change with the time of day and that disruption of the circadian rhythms is linked to inflammatory pathologies. Previous studies have noted the importance of the circadian clock in age-related neuroinflammatory sensitization [27, 28]. BMAL1 is the central mediator of the circadian control of the immune system and anti-inflammatory system, as it drives the expression of *RORα*, which can increase the levels of IκBα [10, 29], a major negative regulator of NF-κB [30]. Furthermore, REV-ERBα is also a transcriptional repressor that inhibits the *BMAL1* expression [26]. LPS-induced sepsis is less severe when injection at ZT10 than at ZT12, coinciding with a decrease in pro-inflammatory cytokines TNF-α, IL-6, and CXCL1 at ZT12, and increase in the anti-inflammatory cytokine IL-10. This effect is blunted a myeloid-specific *BMAL1*^*−/−*^ mouse, indicating the anti-inflammatory role of BMAL1 in myeloid-lineage cells [31]. A recent report has demonstrated that activation of the NF-κB pathway in both primary microglia experiments and *REV-ERBα*^*−/−*^ brain, demonstrating that REV-ERBα negatively controls microglial activation and neuroinflammation [32]. These observations are consistent with the previous observations that the NF-κB-mediated transcriptional repression of the clock feedback limb could cause circadian disruption in response to inflammation [33, 34]. These results suggest that REV-ERBα/BMAL1 is a regulator of microglial activation and neuroinflammation. However, little is known about the impairment of the intrinsic microglial clock in the early phase of AD. Furthermore, the involvement of an altered circadian clock gene expression in the increased presence of microglia with an inflammatory phenotype in AD remains poorly understood. To address these questions, we explored mRNA expression profiles of circadian clock genes and pro-inflammatory genes in cortical microglia isolated from 2-month-old APP-KI and WT mice using a combination of CAGE sequencing and q-PCR analyses. We first found that the mean mRNA expression of *BMAL1* and *REV-ERBα* was significantly lower in APP-KI microglia than in WT microglia. In contrast, the mean mRNA expression of pro-inflammatory genes, including *TNF-α*, *IL-1β*, and *IL-6*, at ZT14 was significantly higher in APP-KI microglia than in WT microglia. It was also noted that the diurnal mRNA expression of these pro-inflammatory genes was negatively associated with that of *BMAL1.* In contrast, the diurnal mRNA expression of *IκBα* was positively associated with that of *BMAL1*. Previous study reported that RORα inhibits NF-κB signaling by inducing IκBα gene expression [29]. We reported that chronic activation of NF-κB promoted increased expression of pro-inflammatory mediators including IL-1β, TNFα, and iNOS [21]. On the basis of these previous observations, it is likely to speculate that reduced expression of BMAL1 may promote the NF-κB activation and the subsequent increased expression of pro-inflammatory genes in APP-KI microglia.

A cross-sectional study had been conducted to examine the association between circadian function, aging, and preclinical AD pathology which included 189 cognitively normal participants. After correction for age and sex, the presence of preclinical amyloid plaque pathology, assessed by positive PiB imaging or increasing cerebrospinal fluid was associated with increased intradaily variability, suggesting preclinical AD is associated with rest-activity rhythm fragmentation and circadian dysfunction could contribute to the early pathogenesis of neurodegenerative disease [35]. In the present study, we used single humanized 2-month-old APP-KI mice. Compared to 6-month- old APP-KI mice, the 2-month-old mice did not exhibit Aβ deposition and activated microglia at the site of Aβ deposition in the hippocampus and cerebral cortex. Therefore, 2-month-old APP-KI mice were reasonable to be used to study the pathogenesis in the early stage of AD.

SR9009, which was developed as a synthetic REV-ERBα agonist, altered the circadian behavior and the circadian pattern of the core clock gene expression in the hypothalamus of mice [36]. Furthermore, LPS-induced IL-6 expression was attenuated in microglia from BMAL1-deficient mice. This phenotype was recapitulated by pharmacological disruption of oscillatory rhythmicity using SR9009 [37]. In a previous study, the REV-ERBα agonists, GSK4112, and SR9001 appeared to prevent neuroinflammation and cell death when infused into the brain [38]. These observations prompted us to examine whether or not the pharmacological activation of REV-ERBα could ameliorate neuroinflammatory response culminating in the disruption of learning and memory. The WT mice showed a response to the novel object and were able to discern a change in the object even after treatment with SR9009. Rather surprisingly, APP-KI mice did not show a response and could not discern a change in the object after treatment with SR9009, suggesting that pharmacological activation of REV-ERBα induced memory disruption in APP-KI mice but not in WT mice. On the other hand, SR9009 has been reported to have REV-ERB-independent proliferation and metabolism [39]. Therefore, additional experiments using REV-ERB deficient microglia are necessary to examine whether a memory impairment effect of SR9009 is due to an activation of REV-ERB.

It is interesting to speculate that OAβ enhances the effects of SR9009, as OAβ is generated even in the brain of 2-month old APP-KI mice. Furthermore, the APP expression is required for microglial activation, particularly in response to OAβ [40]. Our observations in the present study showed that treatment with SR9009 alone was able to increase the expression of *IL-1β* and *IL-6* but not that of *TNF-α* in microglial cells. In contrast, OAβ failed to increase the expression of these pro-inflammatory genes. The combined treatment with SR9009 and OAβ significantly increased the *TNF-α* expression and further enhanced the expression of *IL-1β* and *IL-6* in microglial cells. The present finding that OAβ significantly suppressed *BMAL1* expression in microglia might suggest the possible involvement of decreased the *BMAL1* expression and the subsequent activation of NF-κB in OAβ-induced neuroinflammation. Additional experiments will be needed to confirm this possibility. In the present study, systemic treatment with SR9009 induced activation of hippocampal microglia, increased expression of inflammatory mediators in the hippocampus, and memory disruption in APP-KI mice. SR9009 alone also increased the expression of IL-1β and IL-6 in MG6 microglia. The combined treatment with OAβ and SR9009 further increased expression of these cytokines. In contrast, Griffin et al. have reported that SR9009 exhibited dose-dependent suppression of LPS + ATP-induced IL-1β protein secretion in BV-2 cells and primary cultured microglia [32]. Moreover, an application of REV-ERBα agonist GSK4112 suppressed LPS-induced microglia activation through NF-κB pathway resulting in suppressing the expression and secretion of the pro-inflammatory cytokines [38]. In the present study, however, we could not see anti-inflammatory effects of SR9009. On the contrary, SR9009 induced pro-inflammatory mediators in both cultured microglia and the cerebral cortex of APP-KI mice. This discrepancy may stem from differences in experimental procedures. In our in vitro studies, MG6 microglia were synchronized by treatment with dexamethasone before treatment with SR9009. Dexamethasone was used in the concentration that does not induce anti-inflammatory effects. The inflammatory effect of SR9009 on cultured microglia corresponded well with our in vivo findings.

It has been reported that RORα can exert bi-directional regulation of IL-6 expression dependent on the activation state of astrocytes [41]. RORα may negatively regulate IL-6 expression through the NF-κB pathway in reactive astrocytes. On the other hand, RORα may trans-activate IL-6 expression by interacting with a RORE in the promoter of non-reactive astrocytes. For these two pathways, RORα competes with REV-ERBα that binds the same response elements with a repressor activity. These observations prompted further examination of an anti-inflammatory effect of SR9009 on the strongly activated microglia, because Griffin et al. used a high concentration of LPS (i.e., 50 ng/mL) to activate microglia [32]. As shown in (Additional file 2: Figure S1), SR9009 exerted an anti-inflammatory effect on non-synchronizing and strongly activated MG6 microglia treated with LPS (50 ng/mL). Therefore, it is considered that SR9009 activates NF-kB pathway in synchronizing and mildly activated microglia through activation of REV-ERBα, while SR9009 may repress NF-κB in non-synchronizing and strongly activated microglia by interacting with RORE through activation of REV-ERBα. However, the exact action mechanism of SR9009 on microglia is to be elucidated in future studies.

Several recent reports suggest that circadian dysfunction has a causal role in AD-related neurodegeneration [42–44]. However, neuronal circadian dysfunction alone cannot fully explain the mechanistic relationship between circadian dysfunction and neurodegeneration, because neuroinflammation may play a crucial role in AD-related neurodegeneration [3, 4]. In the present study, we have found that microglia isolated from APP-KI mice exhibited circadian alterations that may be associated with excessive neuroinflammation. Therefore, it may be concluded that circadian disturbances of BMAL1/RORα/NF-κB crosslinking in microglia may contribute to the early stage of AD pathologies through induction of excessive neuroinflammation.

## Conclusion

In summary, we found that microglial clock gene expression was disturbed in the early phase of APP-KI mice. The reduction expression of BMAL1 in the microglia from APP-KI mice at ZT14 or after SR9009 exposure was shown to be responsible for the increased presence of microglia with an inflammatory phenotype through a reduction in RORα, which in turn reduced IκBα and enhanced NF-κB activation. Therefore, microglial clock gene disturbance and resultant chronic neuroinflammation may contribute to the onset of AD.

## Additional files

Additional file 1: **Table S1.** JTK-Cycle analysis for 24h rhythmicity in clock gene expression in microglia. (DOCX 13 kb)

Additional file 2: **Figure S1.** SR9009 suppressed the mean mRNA expression of IL-1β and TNF-α in MG6 microglia without synchronization. (DOCX 587 kb)

## Data Availability

The data used in this study are available from the corresponding authors upon reasonable request.

## Abbreviations

AD: Alzheimer’s disease
ADJ.P: Adjusted *p* value
APP: Amyloid precursor protein
APP-KI: APP knock-in
Aβ: Amyloid beta
BMAL1: Brain and muscle Arnt-like protein-1
C5ar1: COMPLEMENT component 5a receptor 1
CAGE: Cap analysis gene expression
CLOCK: Circadian locomotor output cycles kaput
CLSM: Confocal laser scanning microscope
CPM: Minimum count per million
CSF1R: Colony-stimulating factor 1 receptor
Cxcl3: C-X-C motif chemokine ligand 3
Dex: Dexamethasone
IDR: Irreproducible discovery rate
II1rn: Interleukin-1 receptor antagonist
II1α: Interleukin-1α
IL-1β: Interleukin-1β
IL-6: Interleukin-6
IκBα: Inhibitor of κBα
LPS: Lipopolysaccharide
MACS: Magnetic-activated cell sorting
NF-κB: Nuclear factor-κB
NOS2: Nitric oxide synthase 2
OAβ: Oligomeric Aβ
PER1: Period 1
PER2: Period 2
REV-ERB: Nuclear receptor subfamily1 group D member 1
RORα: Retinoic acid receptor-related orphan receptor α
RT-PCR: Real-time PCR
S100α8: S100 calcium-binding protein A8
S100α9: S100 calcium-binding protein A9
Saa3: Serum amyloid A3
TNFrsfla: Tumor necrosis factor receptor superfamily member 1A
TNF-α: Tumor necrosis factor-α
WT: Wild type
ZT: Zeitgeber time
