# Supplementary material for: An impaired intrinsic microglial clock system induces neuroinflammatory alterations in the early stage of amyloid precursor protein knock-in mouse brain
Source: J Neuroinflammation. 2019 Aug 30;16:173. doi: 10.1186/s12974-019-1562-9 (PMC6716829; doi:10.1186/s12974-019-1562-9)
Supplement: Supplementary file 1 — Table S1. JTK-Cycle analysis for 24h rhythmicity in clock gene expression in microglia. (DOCX 13 kb) [file 12974_2019_1562_MOESM1_ESM.docx]

**Additional file1: Table S1: JTK-Cycle analysis for 24h rhythmicity in clock gene expression in microglia.**

The rhythmicity in clock genes expression in microglia was analyzed by JTK_Cycle analysis. We observed adjusted p-value (ADJ.P) in PER1 of both WT and APP-KI and that in PER2 of WT mice showed < 0.05. However, The ADJ.P in APP-KI mice were all increased compared to that of WT mice in all the analyzed clock genes.

| **GENE** | **GENOTYPE** | **BH.Q** | **ADJ.P** | **LAG** | **AMP** |
| --- | --- | --- | --- | --- | --- |
| **BMAL1** | WT | 1.000 | 0.872 | 0 | 0.162 |
|  | APP-KI | 1.000 | 1 | 10 | 0.047 |
| **PER1** | WT | 0.023 | 0.002 | 16 | 2.185 |
|  | APP-KI | 0.091 | 0.033 | 10 | 1.928 |
| **PER2** | WT | 0.043 | 0.010 | 14 | 1.831 |
|  | APP-KI | 1.000 | 1 | 10 | 0.284 |
| **REV-ERBα** | WT | 0.183 | 0.091 | 12 | 0.285 |
|  | APP-KI | 0.229 | 0.143 | 4 | 0.288 |

Abbreviations: BH.Q, Benjamani-Hochlberq q-value; ADJ.P, Adjust p-value; LAG, phase; AMP, amplitude.
