# Supplementary material for: An impaired intrinsic microglial clock system induces neuroinflammatory alterations in the early stage of amyloid precursor protein knock-in mouse brain
Source: J Neuroinflammation. 2019 Aug 30;16:173. doi: 10.1186/s12974-019-1562-9 (PMC6716829; doi:10.1186/s12974-019-1562-9)

**Additional file2: Figure S1: SR9009 suppressed the mean mRNA expression of IL-1β and TNF-α in MG6 microglia without synchronization**

**A**, **B**, The mRNA levels of IL-1β (A) and TNF-α (B) were quantified using real-time PCR in MG6 microglia treated with 50ng/ml LPS or combination with 10μM SR9009 for 8 h. The asterisks indicate a statistically significant difference from the control group (****P*<0.001, Student’s *t-*test). The daggers indicate a statistically significant difference from LPS group († † *P*<0.01 and † † † *P*<0.001, Student’s *t-*test).


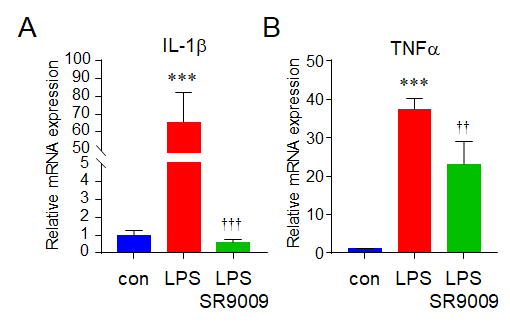

Supplement: Supplementary file 2 — Figure S1. SR9009 suppressed the mean mRNA expression of IL-1β and TNF-α in MG6 microglia without synchronization. (DOCX 587 kb) [file 12974_2019_1562_MOESM2_ESM.docx]
